# Protein A Modulates Neutrophil and Keratinocyte Signaling and Survival in Response to *Staphylococcus aureus*

**DOI:** 10.3389/fimmu.2020.524180

**Published:** 2021-02-22

**Authors:** Camila Ledo, Cintia D. Gonzalez, Ailin Garofalo, Florencia Sabbione, Irene A. Keitelman, Constanza Giai, Inés Stella, Analía S. Trevani, Marisa I. Gómez

**Affiliations:** ^1^ Centro de Estudios Biomédicos, Aplicados y Desarrollo (CEBBAD), Departamento de Ciencias Biológicas y Biomédicas, Universidad Maimonides, Buenos Aires, Argentina; ^2^ Consejo Nacional de Investigaciones Científicas y Técnicas (CONICET), Buenos Aires, Argentina; ^3^ Instituto de investigaciones en Microbiología y Parasitología Médica (IMPaM), Consejo Nacional de Investigaciones Científicas y Técnicas, Universidad de Buenos Aires, Buenos Aires, Argentina; ^4^ Departamento de Inmunología, Instituto de Medicina Experimental (IMEX)-CONICET, Academia Nacional de Medicina, Buenos Aires, Argentina; ^5^ Facultad de Ciencias de la Salud, Universidad Maimónides, Buenos Aires, Argentina; ^6^ Departamento de Microbiología, Parasitología e Inmunología, Facultad de Medicina, Universidad de Buenos Aires, Buenos Aires, Argentina

**Keywords:** *Staphylococcus aureus*, protein A, neutrophils, type 1 TNF-α receptor, cell death

## Abstract

The type 1 TNF-α receptor (TNFR1) has a central role in initiating both pro-inflammatory and pro-apoptotic signaling cascades in neutrophils. Considering that TNFR1 signals *Staphylococcus aureus* protein A (SpA), the aim of this study was to explore the interaction of this bacterial surface protein with neutrophils and keratinocytes to underscore the signaling pathways that may determine the fate of these innate immune cells in the infected tissue during staphylococcal skin infections. Using human neutrophils cultured *in vitro* and isogenic staphylococcal strains expressing or not protein A, we demonstrated that SpA is a potent inducer of IL-8 in neutrophils and that the induction of this chemokine is dependent on the SpA-TNFR1 interaction and p38 activation. In addition to IL-8, protein A induced the expression of TNF-α and MIP-1α highlighting the importance of SpA in the amplification of the inflammatory response. Protein A contributed to reduce neutrophil mortality prolonging their lifespan upon the encounter with *S. aureus*. Signaling initiated by SpA modulated the type of neutrophil cell death *in vitro* and during skin and soft tissue infections (SSTI) *in vivo* triggering the apoptotic pathway instead of necrosis. Moreover, SpA induced pro-inflammatory cytokines in keratinocytes, modulating their survival *in vitro* and preventing the exacerbated necrosis and ulceration of the epithelium during SSTI *in vivo*. Taken together, these results highlight the importance of the inflammatory signaling induced by protein A in neutrophils and skin epithelial cells. The ability of protein A to modulate the neutrophil/epithelial cell death program in the skin is of clinical relevance considering that lysis of neutrophils and epithelial cells will promote an intense inflammatory response and contribute to tissue damage, a non-desirable feature of complicated SSTI.

## Introduction

Neutrophils are essential components of the innate immune response. They constitute an abundant pool in the circulation and they are rapidly recruited into infection sites (1). Neutrophils express a large number of cell surface receptors for the recognition of microbes as well as to sense the inflammatory microenvironment (2). Among this last group are the TNF-receptor family members. TNF-α is a major cytokine triggering neutrophil activation and priming of responses to additional stimuli (3). The type 1 TNF-α receptor (TNFR1) has a central role in initiating both pro-inflammatory and pro-apoptotic signaling cascades in neutrophils depending on the molecules recruited to the receptor (4, 5). In addition to the cognate ligands recognition, TNFR1 signals bacterial products such as the staphylococcal protein A (SpA), a highly conserved surface protein attached to the bacterial cell wall (6). We initially demonstrated that protein A signals through TNFR1 in airway epithelial cells (6). Further research allowed us and other researchers to demonstrate that protein A initiates TNFR1 signaling cascades in other cell types such as monocytes/macrophages (7, 8), kenatinocytes (9), osteoclasts precursors (10) and osteoblasts (11, 12). The interaction of staphylococcal protein A with neutrophils, and the signaling cascades initiated by TNFR1 in these cells, however, has not yet been established.

*Staphylococcus aureus* is an important human pathogen that colonizes the human anterior nares, which represents a major source for invasive infections (13). This microorganism causes a broad range of infections among which skin and soft tissue infections (SSTI) represent a major threat in public health (14). We have recently demonstrated that protein A plays a critical role in cutaneous abscess formation during SSTI (15). In the absence of protein A-mediated inflammatory signaling, neutrophil recruitment to the skin and their focalization within the infected tissue was severely compromised leading to poor abscess formation, deficient resolution of the infection and defective wound healing (15).

Neutrophil lifespan must be strongly regulated to prevent the release of their cytotoxic content into the inflammatory site and therefore, these cells rapidly undergo spontaneous apoptosis (4, 5). However, at inflammatory foci, their apoptotic program can be regulated by a number of agents such as cytokines, pathogens, and environmental stressors (16). In this regard, premature neutrophil apoptosis might result in failure of host defense, whereas a delay in their apoptosis might provide sufficient time for the host to generate defensive responses and/or to induce tissue injury. Given the importance of the modulation of neutrophil lifespan for proper abscess formation (17) the aim of this study was to further explore the interaction of *S. aureus* protein A with neutrophils to underscore the signaling pathways that may determine their fate in the infected tissue during staphylococcal skin infections. In addition, the impact of protein A-mediated signaling in other innate immune cell population critical for the initiation of inflammation in the skin, namely the epithelial cells, was explored.

## Materials and Methods

### Recombinant Proteins and Bacteria

Protein A fused to GST (full length protein A, SpA), the mutated protein A Y14A fused to GST and the GST tag alone were purified as described previously (18). A His-tag protein A was used for certain experiments (19, 20). The recombinant tagged proteins and the GST tag were cleaned of potential lipopolysaccharide contamination using Detoxi-Gel Endotoxin Removing Gel columns (Pierce), dialyzed against endotoxin-free PBS and stored at –80°C until use. The recombinant proteins were used at a concentration of 0.5 µM, unless indicated, for *in vitro* stimulation based on our previous studies (6, 21). The *S. aureu*s strain Newman and the isogenic protein A deficient mutant (SpA^−^) were previously provided by Dr. Tim Foster (Trinity College Dublin, Ireland). Bacteria were grown in Trypticase soy agar (TSA) (Britania, Buenos Aires, Argentina) under conditions that allowed maximal stimulation of each of the cell types used as well as to assure protein A maximal expression. For neutrophil stimulation, bacteria were suspended at OD_600_ = 1 in RPMI 1640 medium (GIBCO-BRL, USA) with 10% FBS (Internegocios SA, Buenos Aires, Argentina) and then diluted to reach a final concentration of 1x10^8^ CFU/ml. For keratinocyte stimulation, bacteria were grown up to OD_600_ = 0.8 and the concentration adjusted in Dulbecco’s modified Eagle’s medium (DMEM, GIBCO-BRL, USA) with 5% FBS according to the multiplicity of infection (MOI) used in each experiment. For *in vivo* inoculation, bacteria were suspended in PBS at a final concentration of 1x10^9^ CFU/ml. The medium generation time and the production of α-haemolysin was equivalent among the wild type strain and the SpA^−^ mutant (15).

### Human Neutrophil Isolation

Blood samples were obtained from healthy donors under informed consent according to the Declaration of Helsinki by venipuncture of the forearm vein and it was drawn directly into heparinized polypropylene tubes. Neutrophils were isolated by Ficoll–Hypaque gradient centrifugation and dextran sedimentation followed by cold hypotonic lysis (Ficoll and dextran, GE Healthcare, Munich, Germany; Hypaque, Winthrop Products, Buenos Aires, Argentina), as previously described (22). Cells were suspended in RPMI 1640 supplemented with 10% FBS at a concentration of 1x10^7^ cells/ml. All neutrophil preparations were analyzed by flow cytometry to guarantee that their Forward Scatter versus Side Scatter (FSC/SSC) parameters were compatible with those of non-activated cells (**Supplementary Figure 1A**). The levels of monocyte contamination were always <0.5%, as evaluated by CD14 (BioLegend) staining and flow cytometry (**Supplementary Figure 1A**). To minimize neutrophil spontaneous activation, cells were used immediately after isolation.

### Neutrophil Stimulation

Neutrophils (10^6^ cells) were seeded in 96 well plates and exposed to recombinant proteins (0.1, 0.5, or 1 µM) or the different strains of *S. aureus* at a MOI of 20:1 (based on previous experiments that showed that 20:1 was the dose of bacteria that induced the most reproducible amounts of IL-8 under the conditions evaluated) in a final volume of 200 µl (5x10^6^ cells/ml). In the internalization experiments, at different time points, cells were washed with PBS and Gentamicin (5 µg/ml) was added to eliminate the remaining extracellular bacteria. Then the cells were washed with PBS, lysed and bacteria were enumerated by serial dilution and plating in TSA. In certain experiments the cells were pre-treated for 30 min at 37°C with the biochemical inhibitors PD98059 (MEK inhibitor V, 25 µM, Calbiochem) or SB203580 (p38 MAP Kinase inhibitor II, 5 µM, Calbiochem) and then exposed to recombinant protein A. For RNA isolation and whole cell lysates 10^7^ cells were used and the stimulations were performed in conical tubes. At the time points indicated cells were harvested for RNA extraction, protein preparations or flow cytometry analysis and the supernatants were saved for cytokine/chemokine detection.

### Keratinocyte Cell Culture and Infection

The keratinocyte cell line HaCaT (23) was cultured in DMEM supplemented with 10% FBS, glutamine (2 mM), penicillin (100 Uml^−1^) and streptomycin (100 Uml^−1^) (GIBCO-BRL, USA). For bacterial infection, cells were plated in 96-well plates (for cytokine detection, 2x10^4^ cells per well) or 12 well plates (for viability assays, 1x10^5^ cells per well). Cells were grown during 48 h to achieve confluence, the media was replaced by DMEM with 5% FBS and 24 h later the cells were stimulated for the times indicated with *S. aureus* Newman or the isogenic SpA^−^ mutant at MOI of 50 and 100 (for cytokine detection) or MOI: 200 (viability assays) in DMEM supplemented with 5% FBS. In separate experiments confluent cells were stimulated for the times indicated with recombinant SpA at different concentrations (0.5; 1; 2.5 and 5 µM).

### Animals and Housing

Mice were obtained from the animal facility of the Department of Microbiology, School of Medicine, University of Buenos Aires (Buenos Aires, Argentina). The procedures involving laboratory animals were approved by the Institutional Animal Care and Use Committee of the School of Medicine, University of Buenos Aires (IACUC Approval number 809/17) and followed internationally accepted guidelines (24). Animals were maintained in a conventional facility, with controlled temperature (22 ± 1°C), controlled humidity (55%), a 12:12 h light/dark cycle and fed *ad libitum*. Procedures were performed in an experimental room within the mouse facility. Mice were euthanized using CO_2_.

### Mouse Model of Skin Infection

BALB/c mice (8 weeks old) were anesthetized with ketamine (100 mg/kg)/xylazine (10 mg/kg) and subcutaneously inoculated in the shaved flank with 100 µl containing 10^8^ CFU of *S. aureus* Newman or the isogenic SpA^−^ mutant (15). The area of the skin lesion was quantified 24 h after inoculation. For neutrophil viability determination, 24 h after the inoculation, the complete abscess was cut in small pieces and treated with a protease cocktail (hyaluronidase 200 U/ml and collagenase II 250 U/ml) for 1 h at 37°C and 5% of CO_2_ atmosphere with slow agitation. Cell suspensions were filtered through a sterile stainless steel mesh, stained for surface expression of Ly6G and their viability was determined by Annexin V/PI (Propidium Iodine) addition and flow cytometry analysis. The abscess area [A = π (L/2) × W/2] was calculated by measuring the lesion dimensions, length (L) and width (W), with a caliper (25–27). For histopathological analysis, skin lesions at day 3 post-inoculation were fixed with 4% formaldehyde and tissue sections stained with Masson’s Trichromic.

### Real Time RT-PCR

RNA from 10^7^ neutrophils was isolated using TRIzol Reagent (Invitrogen). cDNA was made from 1 μg of RNA using M-MLV Reverse Transcriptase (Promega). For quantitative real-time PCR, amplification was performed in an Applied Biosystems thermal cycler. Primers and annealing temperatures used for amplification are listed in **Table 1**. Human β-actin was used as control for standardization.

**Table 1:** Primers used for RT-Real time PCR.

Gen	Primers	T° annealing
β-Actin	Fw-5'-GTGGGGCGCCCCAGGCACCA-3'	60
	Rv-5’-CGGTTGGCCTTGGGGTTCCAGGGGG-3’	
MIP-1α	Fw-5'-AGCTGACTACTTTGAGACGAGCA-3'	62
	Rv-5'-CGGCTTCGCTTGGTTAGGA-3'	
TNF-α	Fw-5'-GGTGCTTGTTCCTCAGCCTC-3'	60
	Rv-5'-CAGGCAGAAGAGCGTGGTG-3'	

### Cytokine Detection

IL-1β, IL-8, and TNF-α were quantified in culture supernatants of neutrophils and IL-1β and IL-6 in culture supernatants of keratinocytes. Enzyme-linked immunosorbent assay using matched antibody pairs from BD Biosciences (IL-8, TNF-α, and IL-6) or R&D Systems (IL-1β) was performed according to the manufacturer’s instructions.

### Quantitation of Apoptosis/Necrosis

Neutrophils, keratinocytes or cell suspensions obtained from murine abscesses (10^6^ cells per reaction tube) were labeled with FITC-conjugated Annexin V (eBioscience) for 15 min at room temperature, subsequently incubated with PI for 15 min at 4°C (BioLegend) and fluorescence was immediately evaluated by flow cytometry using a BD FACSCanto II cytometer. Collected data were analyzed using the FlowJo software. Neutrophils were gated based on their FSC-A/FSC-H profile (28) (**Supplementary Figure 1B**). Annexin V positive cells were considered apoptotic whereas those positive for both, Annexin V and PI staining were considered necrotic. Positive and negative populations for Annexin V and PI were defined based on the staining of live (negative control) and death (positive control) neutrophils (**Supplementary Figure 1B**). Heat shock was used to kill neutrophils to be used as control for positive staining. Negative staining was set with untreated neutrophils immediately after purification.

### Western Blot

Neutrophils (10^7^ cells per condition) were lysed in 200 µl of RIPA buffer containing 150 mM NaCl, 50 mM Tris-HCl pH 7.4, 1% Triton X-100, 1 mM EDTA, 1 mM PMSF, a protease inhibitor cocktail (Sigma-Aldrich, St. Louis, MO), 1 mM sodium orthovanadate and 100 mM sodium fluoride. Proteins were separated on 8% bis-acrylamide gels, transferred to a nitrocellulose membrane, and blocked with 5% milk in TBST (50 mM Tris [pH 7.5], 150 mM NaCl, and 0.05% Tween) for 1 h at room temperature. Immunodetection was performed using antibodies against phospho-erk1/2 or erk1/2 (Santa Cruz Biotechnology, Dallas, TX), phospho-p38 (Thr180/Tyr182) (Cell Signaling Technology) and β-actin (Sigma-Aldrich, St. Louis, MO), followed by secondary antibodies conjugated to horseradish peroxidase (Santa Cruz Biotechnology, Dallas, TX). The blots were revealed by enhanced chemiluminiscence (ECL) in an Image Quant 300 cabinet (GE Healthcare Biosciences, PA, USA) following the manufacturer instructions. Images were analyzed with ImageJ software.

### Statistics

Statistical significance was determined using One-way ANOVA or Two-way ANOVA and Tukey’s multiple comparison test. Proportions were compared using Fisher’s exact test. Comparisons between two groups were done using Mann Whitney test (for non parametric) or Student *t* test (for parametric). Statistical significance was defined as *p <*0.05. Data were analyzed using the Graph Pad Prism software.

## Results

### *S. aureus* Protein A Induces Inflammatory Signaling in Neutrophils

**Figure 1:**
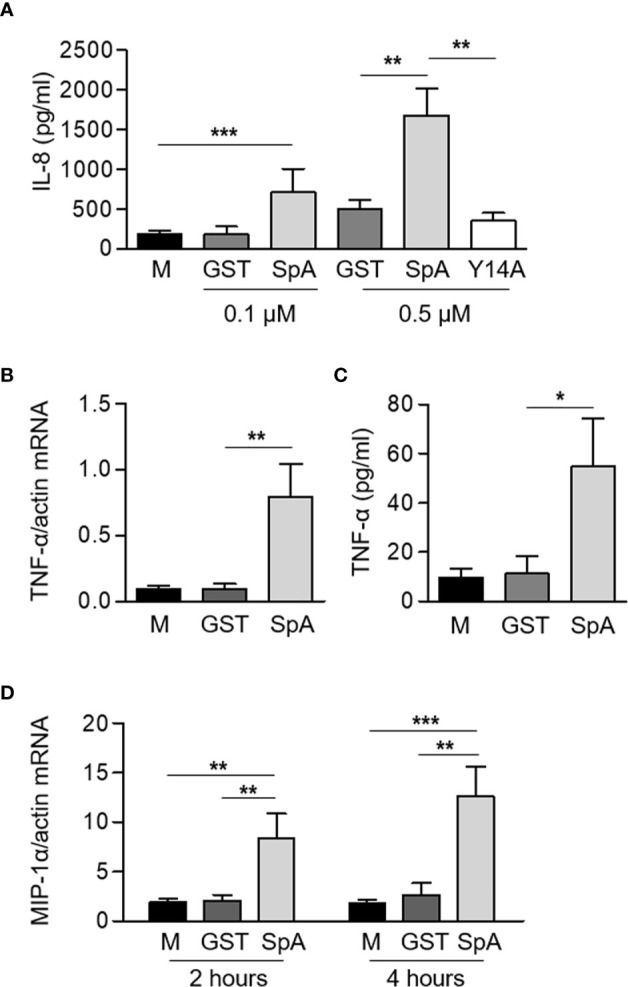
*S. aureus* Protein A induces the production of inflammatory cytokines and chemokines in human neutrophils. Human neutrophils were stimulated for 2 h or 4 h with GST-tagged protein A (SpA) (0.1 µM, panel **A**, 0.5 µM panels **(A–D)** or GST and Y14A-GST (0.5 µM) as control). The production of IL-8 **(A)** and TNF-α **(C)** was quantified by ELISA at 4 h post-stimulation. The levels of expression of TNF-α **(B)** at 2 h post-stimulation and MIP-1α **(D)** at 2 and 4 h post-stimulation were determined by Real Time RT-PCR and standardized by β-actin expression. Bars represent the mean and SEM of six individual donors and three independent experiments. *p < 0.05, **p < 0.01, ***p < 0.001. **(A–C)** One way ANOVA and Tukey’s multiple comparison test; **(D)** Two way ANOVA and Tukey’s multiple comparison test.

We have previously reported the importance of protein A (SpA) in the induction of inflammatory cytokines and chemokines in airway epithelial cells (6), macrophages (7, 8) and osteoclast precursors (10). Neutrophils, although considered for a long time, terminally differentiated immune cells only responsible for phagocytosis are now known to participate in the modulation of the immune response by producing a large plethora of cytokines and chemokines (3). In order to assess whether protein A induces inflammatory signaling in neutrophils, cells were stimulated with purified recombinant SpA. Significant amounts of IL-8 were produced in response to SpA (**Figure 1A**). IL-8 production was not observed when neutrophils were stimulated with Y14A (**Figure 1A**), a mutated SpA unable to interact with TNFR1 (21) confirming that protein A signals through the TNF-α receptor in neutrophils as well as we and other researchers have reported in other cell types (6, 9, 11). A significant increase in the expression of TNF-α at 2 h post-stimulation and the corresponding secretion of this cytokine 2 h later was also observed (**Figures 1B, C**). In addition, SpA induced the expression of MIP-1α at 2 and 4 h after stimulation (**Figure 1D**).

**Figure 2:**
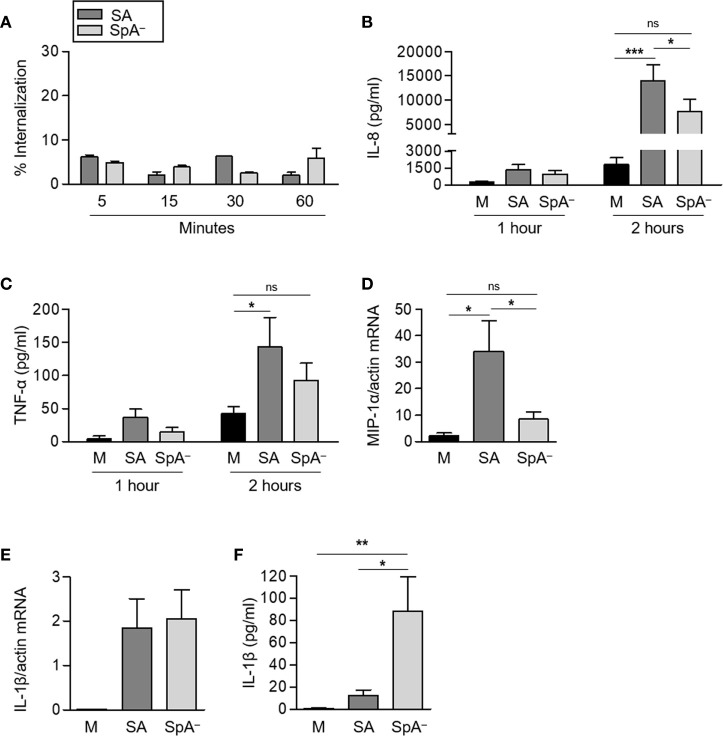
Contribution of Protein A to the induction of pro-inflammatory cytokines and chemokines in response to *S. aureus.* Human neutrophils were stimulated for the times indicated with *S. aureus* wild type (SA) or the isogenic SpA deficient mutant (SpA^−^) at MOI: 20. **(A)** The percentage of intracellular bacteria over a period of 1 h is shown. **(B, C, F)** The production of IL-8 **(B)**, TNF-α **(C)**, and IL-1β **(F)** was quantified by ELISA. **(D, E)** The levels of expression of MIP1-α **(D)** and IL-1β **(E)** at 2 h post-stimulation were determined by Real Time RT-PCR. (Data are expressed relative to β-actin). Bars represent the mean and SEM from six individual donors and three independent experiments. *p < 0.05, **p < 0.01, ***p < 0.001. **(A–C)** Two way ANOVA and Tukey’s multiple comparison Test; **(D)** One way ANOVA and Tukey’s multiple comparison test. ns: not significant.

The biological relevance of the experiments performed using recombinant protein A as stimuli was then evaluated in the context of the whole bacteria using a *S. aureus* mutant that does not express protein A (SpA^−^) in comparison with the isogenic wild type strain. We aimed to explore the signaling cascades elicited by host receptors under conditions resembling those at the initial stages of *S. aureus*-neutrophil contact in the skin tissue. Therefore, the experiments were conducted with non-opsonized bacteria. Under this condition, less than 10% of the bacteria were internalized by neutrophils and no differences in the intracellular content were found between neutrophils stimulated with the wild type bacteria and those challenged with the SpA^−^ mutant over a period of 1 h (**Figure 2A**). IL-8 production in response to *S. aureus* started 1 h post-stimulation and a significant increase in the production of IL-8 was observed at 2 h post-stimulation with *S. aureus* (**Figure 2B**). The SpA^−^ mutant induced lower levels of IL-8 than the wild type strain and statistically significant differences were achieved at 2 h post-stimulation (**Figure 2B**). *S. aureus* also induced the production of TNF-α by neutrophils (**Figure 2C**). Although the levels of this cytokine were also increased in response to the SpA^−^ mutant, no significant differences were observed compared to the control (**Figure 2C**). There was a significant increase in the expression of MIP-1α at 2 h after stimulation with *S. aureus* that was not observed in the absence of SpA expression (**Figure 2D**). Induction of IL-1β transcription in neutrophils was induced by both *S. aureus* and the SpA^−^ mutant at 2 h after stimulation (**Figure 2E**). The levels of detectable IL-1β in the culture supernatant, however, were significantly higher in neutrophils stimulated with the SpA^−^ mutant than those determined in neutrophils stimulated with wild type bacteria. This result is consistent with previous studies in which we demonstrated that wild type *S. aureus* but not the SpA^−^ mutant induce early shedding of the decoy receptor sIL-1RI in monocytes and neutrophils masking the availability of IL-1β (8). Considering the increased mortality induced by the SpA^−^ mutant in neutrophils, the high levels of IL-1β determined could also be due to the release of this cytokine during cell lysis.

### *S. aureus* Induces Pro-Inflammatory Signaling in Neutrophils Through Protein A-Mediated Activation of MAPKs

**Figure 3:**
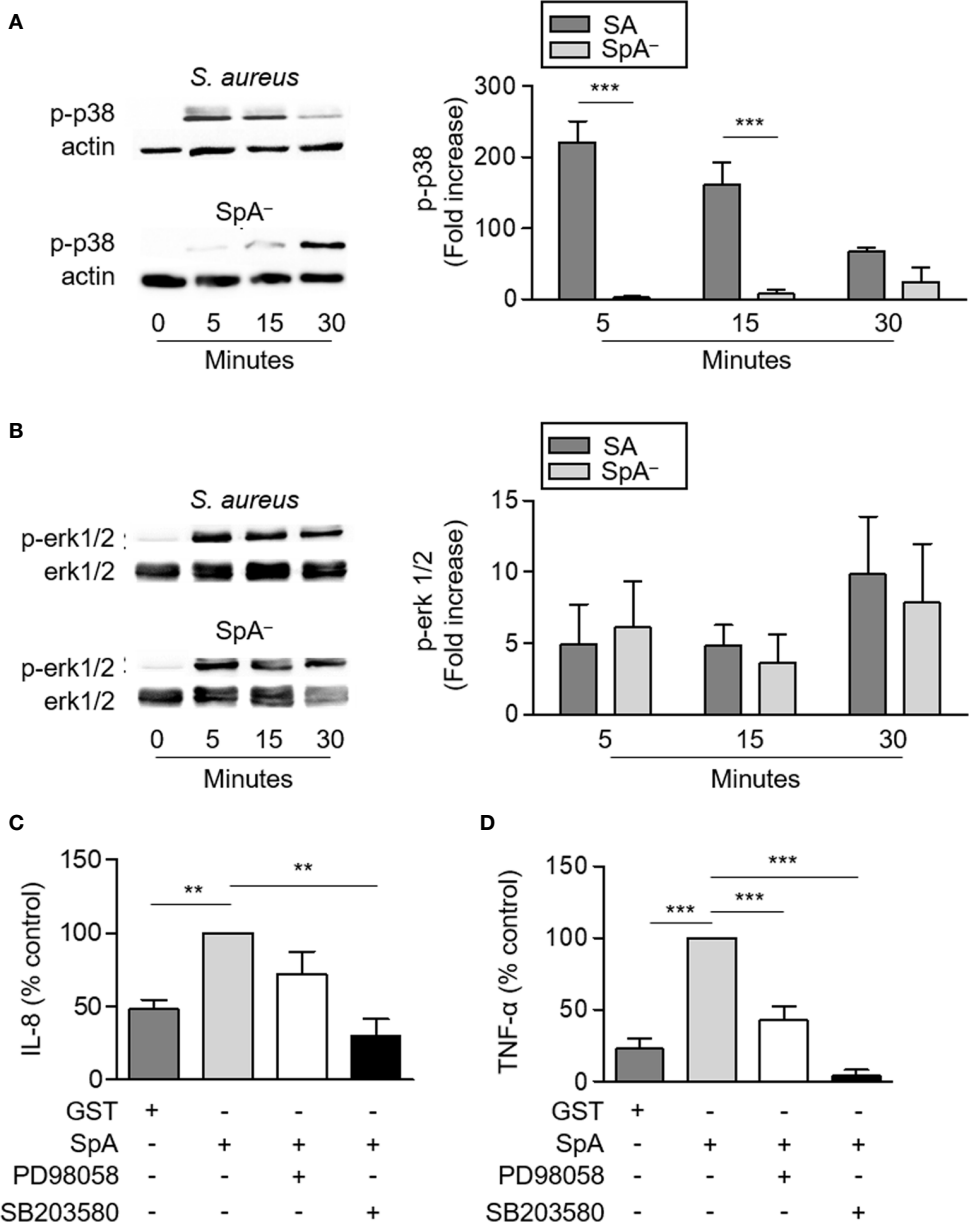
Activation of MAPKs signaling in neutrophils by *S. aureus* Protein A. **(A, B)** Human neutrophils were stimulated with *S. aureus* wild type (SA) or the isogenic SpA deficient mutant (SpA^−^) and the time course of p38 **(A)** and erk1/2 **(B)** phosphorylation was assessed by western blot. Total erk1/2 or β-actin were used as loading control. Bars represent the mean and SEM of the increase at each time point relative to time zero in six individual donors. **(C, D)** Human neutrophils were incubated in the presence or absence of PD98058 (MEK inhibitor; 25 µM) or SB203580 (p38 inhibitor; 5 µM) for 30 min at 37°C and then were stimulated with SpA (0.5 µM) or GST in the presence or absence of inhibitor. The production of IL-8 **(C)** and TNF-α **(D)** was quantified by ELISA at 4 h after stimulation. Bars represents the mean and SEM from six individual donors and three independent experiments. **p < 0.01, ***p < 0.001. **(A, B)** Two way ANOVA and Tukey’s multiple comparison test; **(C, D)** One way ANOVA and Tukey’s multiple comparison test.

TNFR1 signaling induces the activation of p-38 and erk1/2 MAPKs which drive the transcription of pro-inflammatory genes. Therefore, we determined the contribution of protein A to the activation of these MAPKs in neutrophils. *S. aureus* induced phosphorylation of p38 and erk1/2 as soon as 5 min after stimulation (**Figures 3A, B**) as previously reported in airway epithelial cells (6). The SpA^−^ mutant induced significantly decreased levels of p38 phosphorylation at 5 and 15 min post-stimulation compared with the wild type strain highlighting the importance of protein A in the early activation of p38 in neutrophils (**Figure 3A**). Conversely, phosphorylation of erk1/2 was also observed in neutrophils stimulated with the SpA^−^ mutant and no significant differences were quantified compared with the wild type strain (**Figure 3B**), similarly to our previous findings in a different cell type (6). Erk1/2 phosphorylation in the absence of protein A expression is likely due to the action of other inflammatory components of the bacteria such as peptidoglycan and lipoteichoic acids (29). Considering the known importance of MAPKs activation in the induction of inflammatory mediators we then determined whether the production of IL-8 and TNF-α induced by protein A in neutrophils was mediated by p-38 and/or erk1/2 activation. Inhibition of p38, significantly reduced the production of IL-8 and TNF-α by neutrophils in response to protein A (**Figures 3C, D**) whereas inhibition of erk1/2 only blocked the production of TNF-α (**Figure 3D**).

### Protein A-Mediated Signaling Regulates Neutrophil Survival and Type of Death

**Figure 4:**
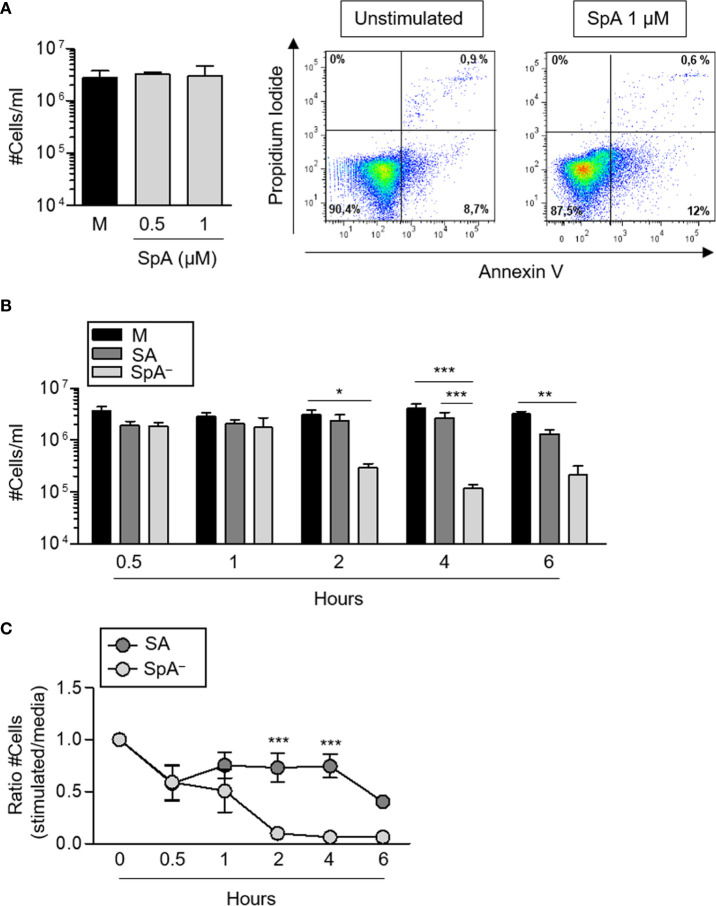
*S. aureus* Protein A modulates neutrophil survival. Human neutrophils were stimulated with His-tagged protein A (SpA) (0.5 µM and 1 µM), *S. aureus* wild type (SA) or the isogenic SpA deficient mutant (SpA^−^) at MOI: 20 for the times indicated. Media alone was used as control. **(A, B)** Cell numbers were quantified for each stimuli. Bars represent the mean and SEM of cumulative data from six donors and three independent experiments. **(A)** Representative plots of neutrophils from one donor before stimulation (left plot) and after stimulation with SpA (right panel). **(C)** Ratio between the number of viable cells enumerated for each stimuli and those enumerated in media at each time point. The mean and SEM is depicted. Cumulative data from six donors and three independent experiments are shown. *p < 0.05, **p < 0.01, ***p < 0.001. **(A)** One way ANOVA and Tukey’s multiple comparison test; **(B, C)** two way ANOVA and Tukey’s multiple comparison test.

**Figure 5:**
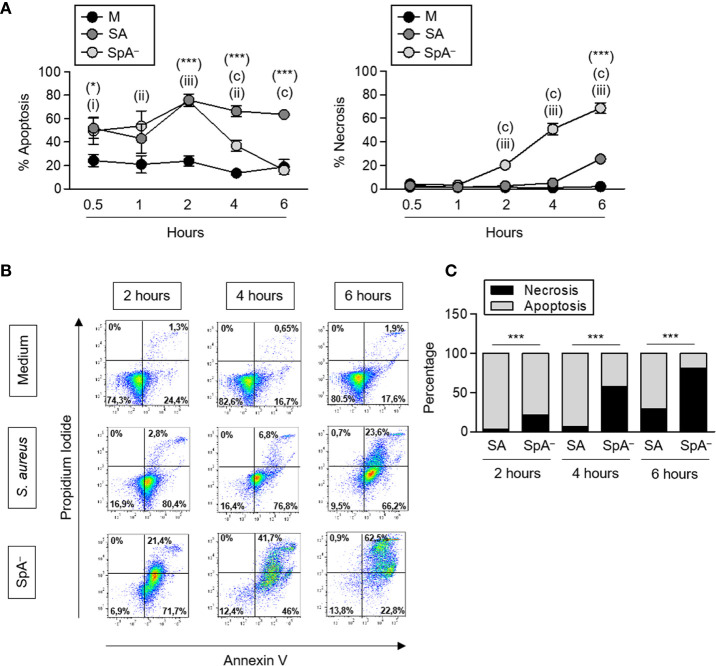
*S. aureus* Protein A modulates neutrophil type of death. Human neutrophils were stimulated with *S. aureus* wild type (SA) or the isogenic SpA deficient mutant (SpA^−^) at MOI: 20 for the times indicated. Media alone was used as control. **(A)** Percentage of apoptotic or necrotic cells were quantified for each stimuli by Annexin V/PI staining and flow cytometry analysis. Circles represent the mean and SEM of cumulative data from six donors and three independent experiments. **(B)** Representative dot plots of Annexin V/PI staining are shown. **(C)** The rate of apoptosis/necrosis for each stimuli was calculated at each time point. Cumulative data from six donors and three in dependent experiments is shown. *p < 0.05, ***p < 0.001, *S. aureus* compared with media; (a): *p* < 0.001, SpA^−^ compared with media; (i):*p* < 0.05, (ii):*p* < 0.01, (iii):*p* < 0.001, *S. aureus* compared with SpA^−^. **(A)** Two way ANOVA and Tukey’s multiple comparison test; **(C)** Fisher’s exact test.

It has been described that certain infectious stimuli can prolong neutrophil survival contributing to bacterial clearance (16). In the case of *S. aureus*, both a delay in apoptosis (30) as well as rapid neutrophil lysis (31) have been reported using different bacterial strains. *S. aureus* produce many virulence factors that contribute to evade phagocytosis. Therefore, to evaluate the impact of early protein A signaling (before phagocytosis can take place) in the modulation of neutrophil survival, cells were stimulated with recombinant SpA, non-opsonized bacteria (wild-type *S. aureus* or the isogenic SpA^−^ mutant) or media alone for different periods of time. Protein A did not induce neutrophil death as assessed by neutrophil counting and Annexin V/PI staining over a period of 2 h (**Figure 4A**). *S. aureus* induced the mortality of neutrophils at early time points after stimulation as we observed a 20% to 40% reduction in the number of neutrophils as soon as 30 min after stimulation with either of the strains evaluated (**Figures 4B, C**). Whereas the number of neutrophils remained stable for the following 3 and a half hours under stimulation with wild type *S. aureus*, in the absence of protein A expression, neutrophil mortality was significantly accelerated (**Figures 4B, C**) suggesting a role for protein A-mediated signaling in prolonging neutrophil survival. In addition to accelerated death, neutrophils stimulated with the SpA^−^ mutant showed increased rates of necrosis/apoptosis compared with cells stimulated with wild type *S. aureus* which were predominantly apoptotic up to 6 h post-stimulation (**Figures 5A–C**). Taken together these results indicate that protein A plays an important role in the induction of pro-inflammatory signaling in neutrophils leading to a reduction in the mortality induced by the bacteria and also modulating commitment of neutrophils to the apoptotic instead of the necrotic death pathway.

### Protein A-Induced Signaling Increases Epithelial Cell Survival During *S. aureus* Infection

**Figure 6:**
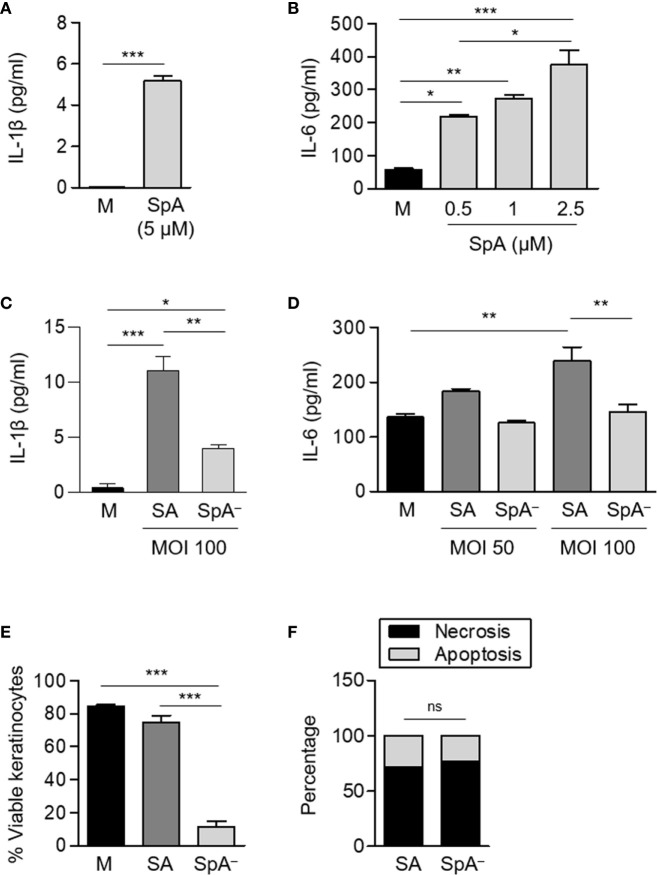
*S. aureus* Protein A induces the production of pro-inflammatory cytokines and modulates keratinocyte survival. **(A–D)** Keratinocytes were stimulated with His-tagged protein A (SpA) **(A, B)**, *S. aureus* wild type (SA) or the isogenic SpA deficient mutant (SpA^−^) at the MOI indicated **(C, D)** and the production of cytokines in the supernatant was quantified at 2 h [IL-6, panels **(B)** and **(D)**] or 4 h [IL-1β, panels **(A)** and **(C)**] post-stimulation. Bars represent the mean and SEM of cumulative data from six wells and three independent experiments. **(E, F)** Keratinocytes were stimulated with *S. aureus* wild type (SA) or the isogenic SpA deficient mutant (SpA^−^) at MOI: 200 for 4 h. Media alone was used as control. **(E)** The percentage of viable cells was quantified for each stimuli by Annexin V/PI staining and flow cytometry analysis. Bars represent the mean and SEM. Cumulative data from six wells and three independent experiments are shown **(F)** The rate of apoptosis/necrosis for each stimuli was calculated and differences were evaluated using Fisher exact test. Cumulative data from six wells and three independent experiments are shown. *p < 0.05, **p < 0.01, ***p < 0.001. **(A–E)** One way ANOVA and Tukey’s multiple comparison test; **(F)** Fisher’s exact test.

We have recently demonstrated that during SSTI the absence of protein A-mediated inflammatory signaling leads not only to poor recruitment of neutrophils and deficient resolution of the infection but also to skin lesions that were significantly increased in size, a phenomenon that it was not dependent on toxin production (15). Therefore, taking into account our findings in neutrophils presented above, we investigated whether protein A also has a role in modulating inflammatory signaling and survival of non professional innate immune cells such as keratinocytes upon *S. aureus* infection. Cells were stimulated with purified protein A, *S. aureus* or the SpA^−^ mutant. Protein A induced IL-1β and IL-6 production in keratinocytes although higher concentrations of SpA were required to stimulate the production IL-1β compare to those required to induce IL-6 (**Figures 6A, B**). The importance of SpA in the induction of these cytokines in the context of bacterial infection was further confirmed by comparing the inflammatory response induced by *S. aureus* and the isogenic SpA^−^ mutant at MOIs that do not induce keratinocyte mortality. Significantly increased levels of IL-1β and IL-6 were induced in keratinocytes stimulated with the wild type strain compared with the protein A deficient mutant (**Figures 6C, D**) confirming the role of SpA in the induction of inflammatory cytokines in keratinocytes. At 4 h after infection at MOI: 200, a significant decrease in viability was observed in cells cultured in the presence of the SpA^−^ mutant compared to those cultured with wild type *S. aureus* (**Figure 6E**). In contrast to the findings in neutrophils, the rate of necrosis/apoptosis did not differ between keratinocytes infected with *S. aureus* expressing or not protein A. In fact, the predominant type of death in both cases was necrosis (**Figure 6F**) indicating that protein A is able to modulate the survival of epithelial cells but cannot change the type of cell death to which they are committed.

### Staphylococcal Protein A-Mediated Signaling Determines the Fate of Neutrophils and Skin Epithelial Cells During *In Vivo* SSTI

**Figure 7:**
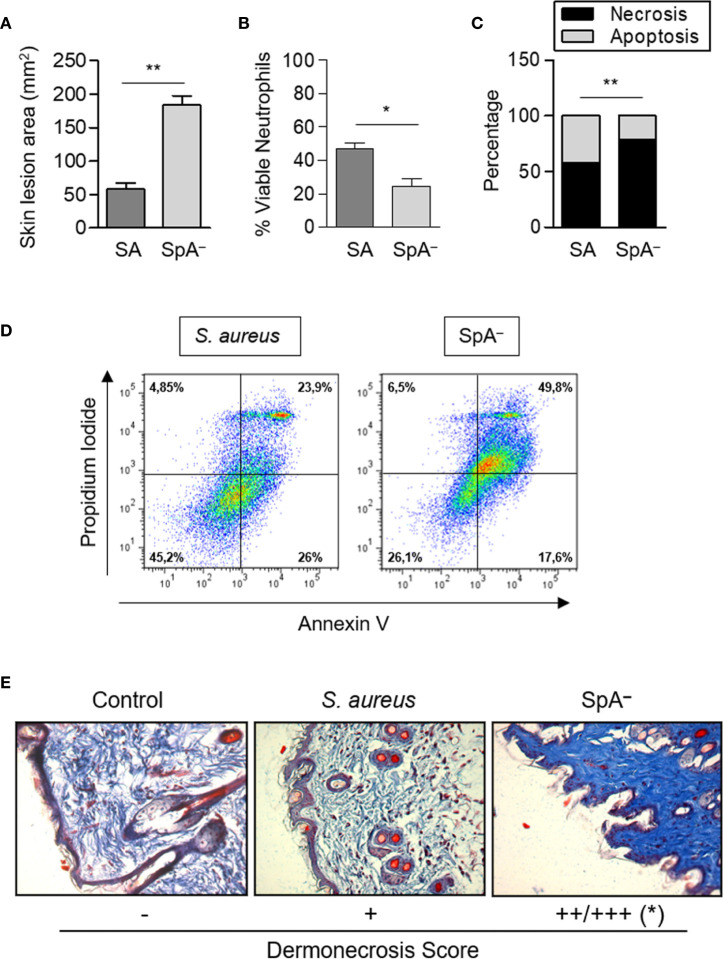
Protein A modulates neutrophil and epithelial cell death pathways *in vivo*. Groups of mice were inoculated with *S. aureus* wild type (SA) or the isogenic SpA deficient mutant (SpA^−^) by the subcutaneous route. **(A)** The area of the skin lesion was determined at 24 h after the inoculation. Bars represent the mean and SEM for each group (n=6 per group). **(B)** Viability of neutrophils present in the skin lesion was evaluated at 24 h post-inoculation by Annexin V/PI staining and flow cytometry analysis. **(C)** The average of the percentage of apoptosis/necrosis in neutrophils obtained from abscess of each group is depicted. **(D)** Representative dot plots of Annexin V/PI staining are shown. **(E)** Masson trichrome staining, 20X magnification. The average of the score assigned to three animals per group is depicted. *p < 0.05, **p < 0.01. **(A, B)** Mann Whitney test, **(C)** Fisher’s exact test and **(E)** Student *t* Test.

Having demonstrated that the inflammatory signaling initiated by protein A modulates neutrophil and keratinocyte survival *in vitro*, we then evaluated whether these findings were relevant to the *in vivo* skin infection. Mice were inoculated by the subcutaneous route with wild type *S. aureus* or the SpA^−^ mutant. The areas of the skin lesion at 24 h post-inoculation were significantly increased in mice inoculated with the SpA^−^ mutant compared with those measured in mice inoculated with *S. aureus* (**Figure 7A**). Neutrophil viability was significantly decreased in the SpA^−^ inoculated mice in relation to the group inoculated with the wild type strain (**Figures 7B, D**), in agreement with our previous findings at a different time point (15). The quantification of apoptosis and necrosis in neutrophils obtained from the abscess showed that, at 24 h after the onset of the cutaneous infection, the rate of necrosis/apoptosis was significantly higher in the abscess from mice inoculated with the SpA^−^ mutant compared with that observed in mice inoculated with wild type *S. aureus*, in which the levels of apoptosis almost equalled those of necrosis (**Figures 7C, D**). These results highlight the importance of protein A-mediated signaling in modulating the type of death in neutrophils *in vivo*. In agreement with the results obtained *in vitro*, protein A expression also modulated necrosis of the epithelium during *in vivo* infection and significantly increased areas of dermonecrosis were observed in mice inoculated with the SpA^−^ mutant compared with mice inoculated with wild type *S. aureus* (**Figure 7E**).

## Discussion

Neutrophils are the first line of defense against invading pathogens. This cell type is, however, often target of several immune evasion strategies that *S. aureus* can present including the modulation of neutrophil cell fate (32–34). Although several types of death have been described after the encounter of *S. aureus* and the neutrophils (32), little is known about the bacterial products responsible for manipulating neutrophil signaling cascades and ultimate cell fate as well as the impact of the infection microenvironment. In this study, we demonstrated that staphylococcal protein A-TNFR1 signaling prolongs neutrophil survival and modulates the type of cell death both *in vitro* and during SSTI *in vivo*. In addition, a role for protein A in preventing epithelial cell necrosis during SSTI is also described.

The majority of the *in vitro* studies aimed to address the modulation of neutrophil death by *S. aureus* have been carried out using bacteria that had been pre-opsonized with autologous serum (30, 31, 35, 36). Under this condition, it has been shown that a low dose of bacteria (MOI: 1) can delay neutrophil apoptosis (30) but also that, in the case of MRSA, phagocytosis of *S. aureus* induces neutrophil lysis within 6 h of the uptake (31). It is important to consider that at the initial stages of infection in certain tissues, such as the skin epithelium, local opsonins may not be efficient to promote phagocytosis due to the large battery of complement inhibitors that *S. aureus* produce including staphylococcal complement inhibitor (SCIN), extracellular complement-binding protein (Ecb) and staphylococcal superantigen-like protein (SSL7) (32, 33, 37). In addition, the immunoglobulin-binding proteins SpA and Sbi (second binding protein for immunoglobulins) are capable of sequestering antibodies (38) further contributing to inhibit phagocytosis. Considering the amount of staphylococcal virulence factors that have the ability to counteract the initial steps of the innate immune response, in particular neutrophil phagocytosis, this study was aimed to explore the signaling cascades elicited by host receptors under conditions resembling those at the initial stages of *S. aureus*-neutrophil contact and, therefore, the experiments were conducted with non-opsonized bacteria.

In addition to the receptors related to phagocytosis, neutrophils express a large number of cell surface receptors involved in the recognition of microbial pathogens as well as in the recognition of the inflammatory environment (2). On the other side, *S. aureus* has the particular characteristic of harboring surface molecules that bind to or signal through host molecules other than conventional PRRs, as it is the case of pore forming toxins such as α-toxin that binds ADAM 10 (39), HlgAB that binds CCR2 and CXCR1/2 (40), HlgCB that binds C5aR (40), Luk ED that binds CCR5 (41), Luk AB that binds CD11b (42),lipoteichoic acids whose recognition through CD36 is required for TLR2 signaling (43) and protein A that signals through TNFR1 and EGFR (6, 44). The fact that *S. aureus* has a surface molecule that signals through the cytokine receptor TNFR1, prompted us to hypothesize that staphylococcal SpA could play a role in the modulation of neutrophil and other innate immune cells survival/death pathways.

We demonstrated here that, similarly to the events reported in airway epithelial cells (6), protein A is also a potent inducer of IL-8 in neutrophils and that the induction of this chemokine was dependent on the SpA-TNFR1 interaction. It is important to consider that in the assays using whole bacteria, the decreased levels of IL-8 observed in neutrophils stimulated with the SpA^−^ mutant may reflect both the capacity of SpA to induce the secretion of this chemokine and also the increased mortality observed in response to the mutant compared to the wild type strain. The pattern of cytokines and chemokines produced by neutrophils depends on the type of stimulus and it is associated to a specific inflammatory context *in vitro* and *in vivo* (45). One of the most potent neutrophil chemoattractant is IL-8 (46). In addition to facilitating neutrophil migration, it can also prime neutrophils for enhanced function. Different staphylococcal molecules like α-toxin (47) and PVL (48) have been shown to induce IL-8 which may explain why in our assays using whole bacteria this chemokine was still produced in the absence of SpA expression. Neutrophil-derived IL-8 can prime the cells in an autocrine fashion and also serve to recruit more neutrophils amplifying the response (3). Priming influences many neutrophil functions including apoptosis (49). The negative correlation between the levels of IL-8 and neutrophil death has been previously reported (36). Therefore, protein A-induced IL-8 may influence the outcome of the *S. aureus*-neutrophil interactions.

Although the production of many cytokines and chemokines by neutrophils has been reported, little is known about the molecular mechanisms controlling their expression in response to *S. aureus*. We demonstrated that protein A-induced IL-8, a cytokine involved in prolonging neutrophil life (36, 50), was dependent on the activation of p38. Moreover, SpA was critical for the phosphorylation of p38, an interesting finding considering that this MAPK has been previously linked to a survival pathway induced by bacterial pathogens in neutrophils (51). In addition to IL-8, we showed that protein A prompted the early induction of MIP-1α, a chemokine involved in the recruitment and activation of neutrophils highlighting the importance of SpA in the amplification of the inflammatory response. *S. aureus* also induced the expression of IL-1β in neutrophils. However, the detectable levels of this cytokine in the *in vitro* assays were very low due to the masking effect of the decoy receptor sIL-1RI previously reported by our group (8).

TNFR1 can mediate both death and survival pathways according to the molecules recruited to the receptor (52). Activation of TNFR1 by its cognate ligand TNF-α, leads to the assembly of a membrane-bound complex that triggers the activation of NF-κB promoting cell survival. In a second step TRADD dissociates from TNFR1 and associates with FADD and caspase-8 to form a cytoplasmic complex which leads to the activation of caspases and cell death (53, 54). In addition, TNFR1 signaling can lead to necroptosis, a programmed form of necrosis, depending on the activation of the RIP-1-RIP3-MLKL pathway (55). The divergent effects of TNF-α on neutrophil survival are related to concentration (56), duration of stimulus (57), and functional state of the cells (58). Whereas concentrations higher than 10 ng/ml enhance apoptosis (59), amounts lower than 1 ng/ml promote NF-kB activation and cell survival (60). Our results indicate that *S. aureus* protein A, contributes to trigger a survival pathway in neutrophils that includes MAPK activation and cytokine/chemokine induction. Although the structure of the protein A-TNFR1 complex has not been elucidated at the molecular level, competition assays showed that TNF-α was able to partially displace SpA from the receptor (6), which suggest that protein A might impact TNFR1 signaling very similarly to the cognate ligand. In agreement with this concept we observed that the amount of TNF-α induced by protein A in neutrophils was within the “pro-survival range” (46). Our findings are in agreement with those of Zurek et al. who demonstrated that programed neutrophil death in response to wild type *S. aureus* was accelerated compared with that observed after the exposure to a *sae*R/S deletion mutant [which it would express higher levels of SpA due to the lack of negative regulation of *spa* (36)]. Other shed bacterial components such as flagellin (61) and LPS (62) as well as secreted toxins can delay neutrophil spontaneous apoptosis increasing neutrophil numbers during early stages of inflammation and providing the appropriate window for clearance of invading microorganisms (16). We provide evidence that staphylococcal protein A is a critical virulence factor that prolongs neutrophil survival. Moreover, the dramatic decrease in cell numbers observed in neutrophils challenged with the SpA^−^ mutant correlates with an increase of a necrotic phenotype as characterized by Annexin V and PI staining. Other bacterial products have been shown to modulate the type of death according to the dose used. Low doses of rPVL (5 nmol/L) induce high levels of IL-8 leading to apoptosis whereas high doses (100 nmol/L) induce necrosis (48).

The biological relevance of the apoptotic program induced by bacterial pathogens in neutrophils is to limit host damage caused by a prolonged inflammatory response. As neutrophils die, their ability to signal in response to ligation of their chemokine and cytokine receptors diminishes. Apoptotic neutrophils still express receptors (non signaling) for these mediators and they can act as an antigenic sink sequestering soluble proteins and precluding them from binding to viable cells (63). Alternatively, during bacterial infections neutrophils can undergo necrosis (primarily due to the action of toxins) (5) or necroptosis (35). Whereas the role of “eat me” signals such as phosphatidylserine expressed by apoptotic neutrophils is a pro-resolving mechanism, necrosis, and necroptosis are largely regarded as pro-inflammatory because the disintegration of the cell membrane leads to the release of cytotoxic neutrophil contents that will damage host cells (63, 64). Our results indicate that protein A not only decreases neutrophil mortality but also contributes to program neutrophils challenged with *S. aureus* to the apoptotic pathway. In this regard, it is of notice that purified protein A induced inflammatory mediators in neutrophils with no associated cell death. Given the importance of TNF-α in the induction of apoptosis, the ability of protein A to signal through TNFR1 is likely to be critical instructing neutrophils to undergo apoptosis upon the encounter with *S. aureus* highlighting the importance of studying virulence factors in the context of the whole bacteria. Although preliminary experimental data seem to suggest that both the RIP1 kinase and caspase-1 might participate to some extent in the death of neutrophils induced by *S. aureus* that does not express protein A (data not shown), further studies will be required to elucidate in detail the signaling cascades involved.

Taking our results together with those of other researchers it is possible to conclude that the interaction of *S. aureus* and neutrophils *in vitro* can lead to distinct paths such as delayed apoptosis [this study and (30)], programmed necrosis (35) or lytic deaths such as cytolysis due to bacterial toxins (5) or NETosis (16) depending upon the strain used and whether the bacteria has been pre-opsonized. All these mechanisms are likely to occur *in vivo* and depending on the type of infection, the infecting strain and the local microenvironment where the infection takes place certain process will be prevalent over others. Therefore, we aimed to determine the role of protein A-TNFR1 signaling in modulating neutrophil cell fate *in vivo* using a mouse model of skin and soft tissue infection previously described (15). Despite the mortality observed at 24 h after the onset of SSTI, protein A expression significantly increased neutrophil survival *in vivo*. Moreover, equivalent to the results obtained *in vitro*, SpA contributed to reduce neutrophil necrosis in the skin increasing the percentage of neutrophils undergoing apoptosis. We have recently demonstrated the importance of protein A expression in the induction of a controlled inflammatory milieu in the skin that leads to proper abscess formation and bacterial clearance (15). In this study we have extended our observations and described the role of protein A in the regulation of neutrophil cell death pathways. In addition, our data shows that protein A also modulates keratinocyte survival thus preventing the exacerbated dermonecrosis and ulceration during SSTI. The ability of protein A to modulate the neutrophil/epithelial cell death program in the skin is of clinical relevance considering that lysis of neutrophils and epithelial cells will promote an intense inflammatory response and contribute to tissue damage, a non desirable feature of complicated SSTI (65–67).

*S. aureus* has evolved to have many virulence factors involved in immune evasion and among them is protein A. This protein contributes to subvert the immune system not only by its very early described role in the inhibition of phagocytosis but also by its more recently defined induction of cytokine receptor and decoy receptor shedding, such as the sTNFR1 and sIL1RI, in several cell types including neutrophils (7, 8). Staphylococcal protein A is certainly an intriguing virulence factor that in addition to immune evasion acts as a highly pro-inflammatory molecule due to its ability to interact with TNFR1 (6, 68). Inflammation is a key component of the innate immune response that has to be tightly regulated. Whereas it is necessary for the eradication of invading microorganisms, if not controlled, it might have deleterious consequences for the host. In this context, whereas protein A-mediated inflammation will constitute an advantage to the bacteria in certain host microenvironments such as the lungs or the bone contributing to the development of pneumonia and osteomyelitis, respectively (6, 10) it might be advantageous for the host in other body compartments such as the skin where it contributes to proper abscess formation and bacterial clearance (15). During the specific interaction with neutrophils described in this work, the dual properties of protein A are also revealed. It has the ability to prolong the life spam of neutrophils as a consequence of its pro-inflammatory potential and at the same time is able to shift the death program to apoptosis, a non inflammatory death pathway. The impact that bacteria may have on neutrophil fate can be beneficial or detrimental to the host depending on the specific microorganism and the infection microenvironment. Elucidating the differential roles that different staphylococcal virulence factors may play in the modulation of innate immune cell survival *in vivo* is critical for the understanding of *S. aureus* pathogenesis and the rational design of alternative therapeutic approaches.

## Data Availability Statement

The data sets generated for this study are available on request to the corresponding author.

## Ethics Statement

The procedures involving laboratory animals were approved by the Institutional Animal Care and Use Committee of the School of Medicine, University of Buenos Aires (IACUC Approval number 809/17). Blood samples were obtained from healthy human donors under written informed consent.

## Author Contributions

MIG, CL, and CDG conceived and designed the experiments, analyzed the data, and wrote the manuscript. CL, CDG, AG, FS, IAK, CG, and IS performed the experiments. AST analyzed the data and critically revised the manuscript. MIG and AST procured funding. All authors contributed to the article and approved the submitted version.

## Funding

This work was supported by grants from the Agencia Nacional de Promoción de la Ciencia y la Tecnología, Argentina (PICT2011-2263, PICT2016-2678 and PICT2018-3613 to MIG; PICT2013/2177 and PICT2016/1418 to AST) and the Secretaría de Ciencia y Técnica, Universidad de Buenos Aires, Buenos Aires, Argentina (UBACyT 20020150100114BA to MIG and UBACyT 20020130100744BA to AST). Intramural funding has been provided by the Fundación Científica Felipe Fiorellino, Buenos Aires, Argentina.

## Conflict of Interest

The authors declare that the research was conducted in the absence of any commercial or financial relationships that could be construed as a potential conflict of interest.

## Supplementary Material

Supplementary Figure 1
**(A)** The FSC/SSC profile of purified neutrophils is shown on the left. The level of monocyte contamination, as evaluated by CD14 staining and flow cytometry is shown on the right. **(B, C)** Neutrophils were gated based on their FSC-A/FSC-H profile to exclude particles/debris/apoptotic bodies (FSC^low^) and doublets (28). Positive and negative populations for Annexin V and PI were defined based on the staining of live (negative control) and death (positive control) neutrophils. **(B)** Negative staining was set with untreated neutrophils immediately after purification. **(C)** Positive staining was set with neutrophils killed by heat shock.
