# Supplementary figures and images for: Protein A Modulates Neutrophil and Keratinocyte Signaling and Survival in Response to Staphylococcus aureus
Source: Front Immunol. 2021 Feb 22;11:524180. doi: 10.3389/fimmu.2020.524180 (PMC7937904; doi:10.3389/fimmu.2020.524180)

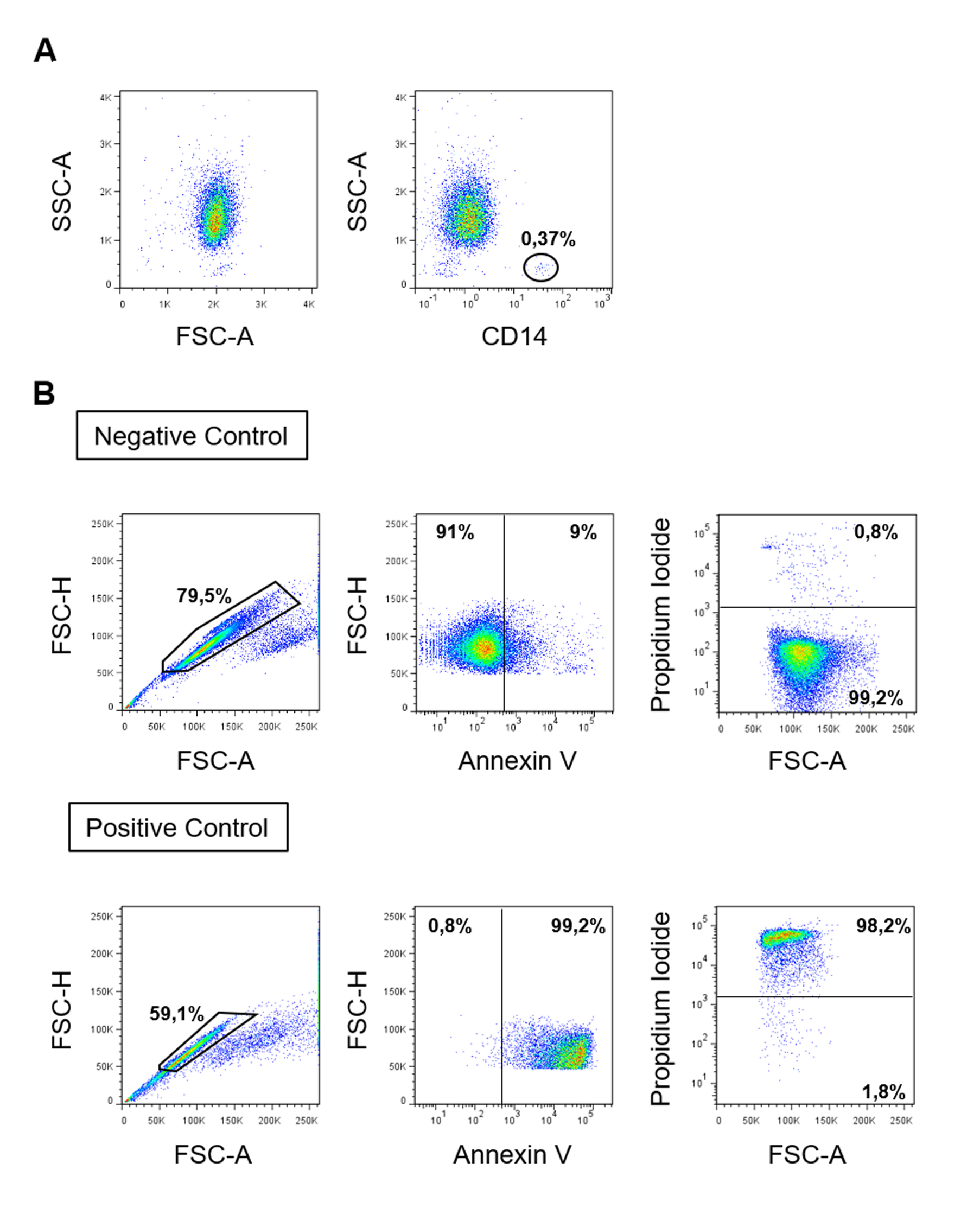

Supplement: Supplementary Figure 1 — (A) The FSC/SSC profile of purified neutrophils is shown on the left. The level of monocyte contamination, as evaluated by CD14 staining and flow cytometry is shown on the right. (B, C) Neutrophils were gated based on their FSC-A/FSC-H profile to exclude particles/debris/apoptotic bodies (FSClow) and doublets (28). Positive and negative populations for Annexin V and PI were defined based on the staining of live (negative control) and death (positive control) neutrophils. (B) Negative staining was set with untreated neutrophils immediately after purification. (C) Positive staining was set with neutrophils killed by heat shock. [file Image_1.tif]
